# Macrophage Activation Syndrome Presents as Initial Manifestation of Lupus in an Adult Female

**DOI:** 10.7759/cureus.60567

**Published:** 2024-05-18

**Authors:** Ahmed H Abdelfattah, Alexandra Macpherson, Faiza Javed

**Affiliations:** 1 Internal Medicine/Hospital Medicine, University of Kentucky College of Medicine, Lexington, USA

**Keywords:** systemic lupus erythematosus (sle), hematology-oncology, hemophagocytic lymphohistiocytosis (hlh), internal medicine and rheumatology, macrophage activation syndrome (mas)

## Abstract

Systemic lupus erythematosus (SLE) is an autoimmune disease characterized by the immune system erroneously attacking healthy tissues and organs. SLE has a wide variety of clinical presentations. The signs and symptoms of SLE are very well-known, though rare presentations could occur that require early clinical attention. Macrophage activation syndrome (MAS) is a severe and life-threatening condition in which the immune system becomes overactive, leading to the excessive stimulation and proliferation of immune cells. MAS can occur as a primary immune disorder, which is not very common. It can also happen secondary to a wide variety of pathological conditions, which include infections, malignancies, autoimmune, and rheumatologic disorders. In rare cases, SLE can present with overlapping features of MAS, further complicating the clinical picture, and may require specialized management. Early recognition and intervention of this overlap are essential for improving outcomes, as delayed diagnosis and treatment can lead to significant morbidity and mortality. Here, we present a case of a young adult female who was diagnosed with SLE with the initial presentation of MAS in the form of fever, splenomegaly, cytopenia, and hemophagocytosis.

## Introduction

Macrophage activation syndrome (MAS), or hemophagocytic lymphohistiocytosis (HLH), is an aggressive immune condition rarely occurring in adults. MAS is characterized by uncontrolled production of proinflammatory cytokines in a condition known as hypercytokinemia [1]. It presents as a primary hereditary immune disorder or can be secondary to various disorders such as infections, malignancies, and autoimmune diseases [1]. Because of their striking similarities in clinical presentation, acute systemic lupus erythematosus (SLE) and acute HLH are difficult to distinguish from one another. Both can present with fever, pancytopenia, high inflammatory markers, coagulopathy, liver affection, lymphadenopathy, high ferritin, and splenomegaly [2]. On rare occasions, SLE can present with HLH, and it is called acute lupus haemophagocytic syndrome (ALHS), which has an incidence of 4.6% according to one study [2]. Thus, it is crucial to make an early diagnosis, as MAS, which is secondary to SLE, responds quickly to steroids and immunosuppressants.

## Case presentation

A woman in her late teens with no past medical history presents with persistent nausea, poor oral intake, blurred vision, and subjective fevers for the last week. She has had ongoing fatigue and malaise with diffuse morning joint stiffness and muscle stiffness for about eight months. Three weeks ago, she presented to her primary care physician a non-tender, nonpruritic, petechial rash on her ankles and feet, slowly progressing to both arms and legs, associated with bilateral lower extremity edema. Her family history is significant for rheumatoid arthritis in her mother and systemic lupus erythematosus in two of her maternal aunts and paternal grandmother.

On arrival at the emergency department, she was febrile (temperature: 38.3 °C) with a heart rate of 120 b/m. Laboratory workup was significant for normocytic anemia with hemoglobin of 9.4 g/dL, thrombocytopenia of 83 x 10^3^/uL, leukopenia 2.5 x 10^3^/uL, acute kidney injury (AKI) with creatinine at 1.5 mg/dL (baseline creatinine: 0.8 mg/dL), acute phase reactant protein (CRP) of 14.8 mg/dL, ferritin of 1,800 ng/mL, normal bilirubin level, serum aspartate aminotransferase (AST) of 55 U/L, alanine aminotransferase (ALT) of 80 U/L, and alkaline phosphatase of 35 U/L. Complete infectious work was pursued, which included fungal, bacterial, and parasitic serologies, comprehensive gastroenteritis panels, mycobacterial cultures, and urine studies. Empirically, the patient was started on piperacillin and tazobactam. Chest imaging showed small bilateral pleural effusions. Abdominal imaging showed moderate diffuse hepatic cirrhosis and iron deposition, splenomegaly, and significant ascites. We performed paracentesis, and peritoneal fluid analysis was notable for serum ascites albumin gradient (SAAG) of <1.1. There was no evidence of infection or malignant cells in the ascitic fluid. We further investigated chronic liver disease by sending workups for autoimmune diseases and Wilson disease. This included positive antinuclear antibody: 1:2650, anti-dsDNA >1:2560, anti-Smith antibodies: 101 units (0-19 units), anti-smooth muscle antibody: 107 units, antineutrophil cytoplasmic antibody (ANCA)/myeloperoxidase (MPO): 48 AU/ mL (0-19 AU/mL), antimitochondrial antibodies: negative, liver kidney microsome antibodies: negative, complement C3: <4 mg/dL, complement C4: <10 mg/dL, activated partial thromboplastin time (aPTT) lupus anticoagulant sensitive: 44 seconds (<=41.0 sec), serum soluble interleukin 2 receptor: 4,469.6 pg/mL (175.3-858.2 pg/mL), plasma triglycerides: 281 mg/dL (<150 mg/dL), serum fibrinogen: 122 mg/dL (208-459 mg/dL), ceruloplasmin: 10 mg/dL (20-60 mg/dL), 24-hour urine copper: 49 ug/dL (3-35 ug/dL), and negative hepatitis panel. A liver biopsy showed moderate fatty changes only. The ophthalmologic evaluation did not reveal Kaiser Fleischer rings. The patient continued to be febrile with empiric antibiotic coverage, so we stopped antibiotics on day five.

**Figure 1:**
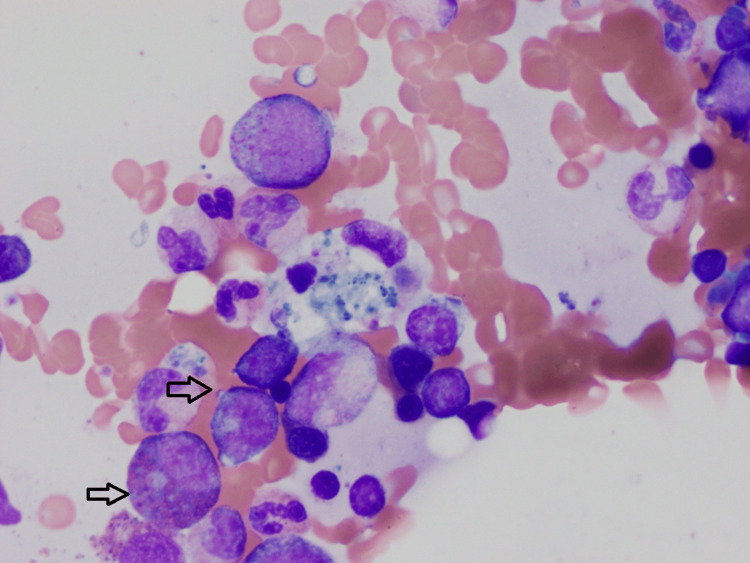
Bone marrow biopsy showing hypocellular marrow for the age (60%) with maturing trilineage hematopoiesis and occasional hemophagocytic histiocytes Black arrows are pointing to hemophagocytic histiocytes.

Lupus was diagnosed based on a prior history of inflammatory arthralgia, skin rashes in sun-exposed areas, serositis, pancytopenia, AKI with non-nephrotic range proteinuria, positive serologies ANA/anti-DNA/Smith. A renal biopsy showed diffuse lupus nephritis ISN/RPS (International Society of Nephrology/Renal Pathology Society) class IV. A bone marrow biopsy showed hypocellular marrow for the age (60%) with maturing trilineage hematopoiesis and occasional hemophagocytic histiocytes (Figure 1).

She was diagnosed with HLH based on 7/9 criteria in adults. This included fever (peak temperature of > 38.5 °C for > seven days), splenomegaly, cytopenia involving > two cell lines, hemophagocytosis (in biopsy samples of the bone marrow), low natural killer cell activity, serum ferritin >500 ng/mL, and elevated soluble interleukin-2 (CD25) levels.

**Figure 2:**
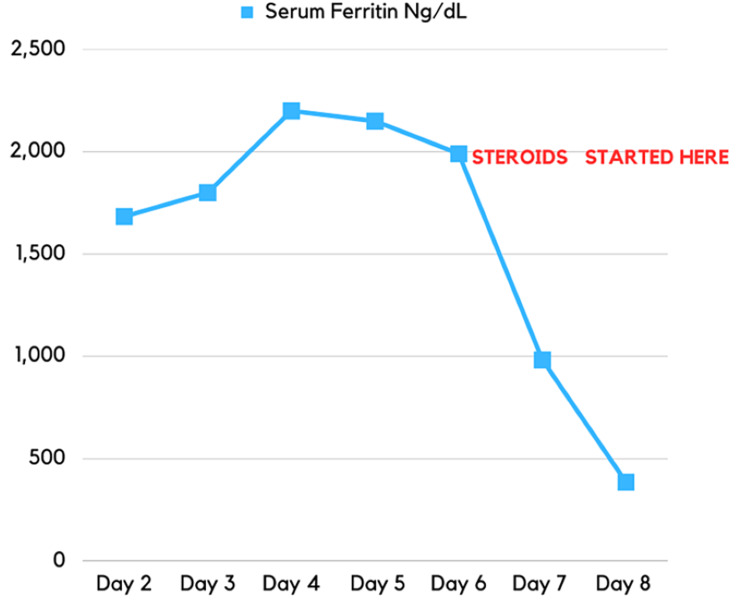
Trend of the serum ferritin during the hospitalization

We started her on dexamethasone 20 mg orally daily per HLH protocol. Liver, kidney function, and ferritin levels slowly improved after the initiation of steroids (Figure 2).

To treat lupus, she was initiated on Plaquenil and mycophenolate orally. She continued to improve clinically and was discharged home with a steroid taper. Three weeks later, the patient presented to the clinic. She denied any complaints. Laboratory tests showed normal liver and kidney function.

## Discussion

MAS is a form of HLH secondary to rheumatic diseases and is characterized by the presence of hypercytokinemia leading to inflammation and organ dysfunction leading to multi-organ failure. Although poorly understood, pathogenesis is secondary to hyper-stimulation of macrophages and cytotoxic T lymphocytes (CD8+ T cells), resulting in a cytokine storm. Some cytokines that have been implicated in pathogenesis are interleukin-2 (IL-2), interleukin-6 (IL-6), interleukin-18 (IL-18), and interferon-gamma (IFN-γ) [1].

MAS/HLH are cytokine storm syndromes (CSS) that are associated with various infections, rheumatic conditions, and hematologic malignancies (among other etiologies). The current diagnostic criteria for HLH/MAS employs the use of five out of nine of the following findings: fever ≥ 38.5 °C; splenomegaly; cytopenia in ≥ two blood lineages; hypertriglyceridemia; hypofibrinogenemia; hemophagocytosis in the bone marrow, spleen, lymph node, or liver, ferritin > 500 ng/mL (although >3,000 ng/mL is more indicative of HLH); low or absent NK elevated sCD25; and elevated CXCL9 [2].

The patient met the 5/8 HLH/MAS criteria. MAS has been reported in various rheumatological diseases, including systemic juvenile idiopathic arthritis, adult-onset still's disease, rheumatoid arthritis, Sjögren's syndrome, dermatomyositis, mixed connective tissue disease, systemic sclerosis, and SLE. Here, we are reporting a case of MAS as the onset of SLE, which is exceedingly rare and has an incidence rate of about 0.9-4.6% [3]. However, there are various cases of MAS associated with SLE flare or its complications. Very few cases of MAS have been reported as the onset of SLE [4-8]. Not only is this presentation rare, but it also has various diagnostic complications.

There are several overlapping features between SLE flare and MAS. We recommend starting with extensive infectious disease work, including bacterial, viral, and fungal etiologies. If infectious workups are inconclusive or if inflammation persists even after treatment, it should raise suspicion of MAS/HLH. We noticed in our patient that she continued to have a fever with elevated inflammatory markers after multiple days of antibiotics. She had a history of inflammatory arthralgia, skin rashes in sun-exposed areas, serositis (peritonitis), pancytopenia (with some labs supporting marrow suppression such as low reticulocyte count), AKI with non-nephrotic range proteinuria, and positive serologies ANA/anti-DNA/Smith antibodies, which were consistent SLE. However, fever with hepatosplenomegaly and lymphadenopathy raised suspicion for MAS and prompted us to a bone marrow biopsy consistent with hemophagocytosis. A diagnosis of MAS should be suspected in an SLE patient with prolonged high fever, peripheral cytopenia, and liver failure. Collecting bone marrow aspirates is critical for accurate diagnosis [9].

Hyperferritinemia is considered the best parameter to discriminate between MAS-associated SLE and active SLE, with a sensitivity and specificity of almost 100% in prior studies [3]. Early recognition of MAS with prompt administration of immunosuppression is critical and lifesaving. Limited data are available from case series/reports for managing macrophage activating syndrome. To our knowledge, no trials are present in the literature. High-dose corticosteroids with immune suppressive therapy targeting various cytokine storm levels seemed beneficial [10].

Glucocorticoids in the form of pulse methylprednisolone are the first choice of treatment. Our patient responded clinically within 48 hours to steroids with a resolution of fever and down-trending inflammatory markers. An intravenous methylprednisolone pulse therapy (e.g., 30 mg/kg for three consecutive days), followed by 2-3 mg/kg per day, is the most common schedule. In nonresponsive cases, the HLH 94 protocol is used with the combined use of steroids, cyclosporine, and etoposide [11]. However, due to the clinical improvement seen in our patient, chemotherapy use seemed unnecessary. Other agents in the literature in non-responsive patients include IL 6 receptor antagonists, Janus kinase (JAK) inhibitors, anakinra, and rituximab [12-14]. We started our patient on hydroxychloroquine and mycophenolate for the treatment of SLE and lupus nephritis.

## Conclusions

The incidence of MAS presenting as an initial feature of SLE is extremely rare. A thorough workup including infectious and malignancy investigations is needed, and the presence of fever, hepatosplenomegaly, and lymphadenopathy with elevated inflammatory markers should prompt a workup of HLH/MAS in lupus patients. Early recognition of MAS with prompt administration of immunosuppression is critical and lifesaving.
